# The aesthetic, artistic and creative contributions of dance for health and wellbeing across the lifecourse: a systematic review

**DOI:** 10.1080/17482631.2021.1950891

**Published:** 2021-08-03

**Authors:** Kerry Chappell, Emma Redding, Ursula Crickmay, Rebecca Stancliffe, Veronica Jobbins, Sue Smith

**Affiliations:** a Graduate School of Education, University of Exeter, Exeter, UK; b Dance Science Department & Learning and Participation Programme, Trinity Laban Conservatoire of Music and Dance, London, UK; c Dartington Trust, Totnes, UK

**Keywords:** Dance for health, participatory dance, systematic literature review, dance and wellbeing, dance health methodologies

## Abstract

**Purpose:**

This review articulates current understanding of the aesthetic, artistic and creative contributions that Dance makes to Health and Wellbeing across the lifecourse within publications 2000–2019, an under-researched area.

**Methods:**

Review Questions: What are the aesthetic, artistic and creative contributions that Dance makes to Health and Wellbeing across the lifecourse? And what methodologies are appropriate for investigating these contributions? A database keyword search identified 769 articles and 91 evaluations. 109 documents were identified for further in-depth analysis and rating, resulting in 24 papers (11 articles, 3 PhD studies, 10 evaluation reports), which were thematically analysed.

**Results:**

Findings offer seven interrelated contributions that Dance makes to Health and Wellbeing: embodiment, identity, belonging, self-worth, aesthetics, affective responses and creativity. There was less insight regarding different methodologies, and discussions focused on quantitative data’s limitations. There were insights into inclusion of embodied voices, subjective accounts, and lived experiences.

**Conclusion:**

Whilst acknowledging challenges, this paper illuminates the key contributions of dance to arts and health. It provides a future conceptual research agenda (prioritizing identity and creativity) and associated methodological developments. It recommends expanding geographical/lifecourse research, better defining terms, fuller epistemological critiques to open space for new methodologies, and continued attendance to appropriate rigour criteria.

## Introduction

The arts, including dance, are increasingly articulated in contemporary society as “supporting specific health conditions and more generally enhancing wellbeing, health behaviours, and social engagement” (Fancourt, 2017, p. 41). It is also argued that participation in the arts as a supplement to medicine and social care can dramatically improve quality of life (Staricoff, 2006; Zeilig et al., 2009).

Research has demonstrated the benefits of dance on improving physical aspects of health and fitness including flexibility, balance and cardiovascular fitness in young people (e.g., Burkhardt & Brennan, 2012; Connolly et al., 2011) and among populations with health conditions (e.g., dos Santos Delabary et al., 2017; Hackney & Earhart, 2009). The impact of dance on psychosocial wellbeing including body image and self-perception has also been documented (e.g., Burgess et al., 2006; Burkhardt & Brennan, 2012). However, despite the field gathering some momentum (Vella-Burrows et al., 2017), understanding of the aesthetic, artistic and creative (as opposed to the purely physical outcomes) contributions that dance makes to health and wellbeing is under-researched and not well understood (Houston & McGill, 2013; Urmston, 2018, 2019; Wakeling & Jenkins, 2019). Not only this, the research field is lacking in terms of an evaluation of appropriate methodologies for investigating these contributions, and their impacts (Camic et al., 2018; Connolly et al., 2011; Hackney et al., 2007; Moss et al., 2015; Quin et al., 2007). The literature review team have collaborated on practice, evaluation and research in dance education and health settings over a period of 20 years (e.g., Chappell et al., 2011; Chappell & Stancliffe, 2018; Redding et al., 2016, 2011) and have increasingly become aware of this twin gap.

This systematic literature review draws together and maps understanding which has particularly blossomed in the last 10 years across disparate elements of dance, health and wellbeing practice and research. It aims to build on reviews such as that by Sheppard and Broughton (2020)—who synthesize understanding of how active participation in music and dance promotes wellbeing and health—by specifically investigating the aesthetic, artistic and creative contributions of dance per se. The review is part of a wider project, *Dance, Health and Wellbeing: Debating and moving forward methodologies* funded by the University of Exeter Wellcome Centre for the Cultures and Environments of Health, which explores how a broad array of stakeholders including academics, health and arts professionals and participants understand and experience the creative, artistic and aesthetic contributions of dance, and how they are researched. The literature review therefore sits alongside and in conversation with a programme of stakeholder workshops exploring these themes, and is only one part of understanding and evaluating practice and its impact, alongside, for example, other types of writing, such as that of Tufnell (2017) and Halprin (2002), who might be described as researchful practitioners and artists. As Daykin et al. (2017) argue, there is a need for more co-production between stakeholders to strengthen evaluation practice and support the development of the arts and health sector; this literature review aims to contribute to this co-production.

Supported by these conversations, in preparing the research questions to drive the review, the following key terms continually presented themselves, with their definitions ultimately shaping the review process: dance, aesthetic, artistic, creative/creativity, health and wellbeing, dance for health.

The review adopts a broad definition of dance, understanding it as expressive human movement with aesthetic and artistic value, often accompanied by music, that can be both performative and participatory, and that is facilitated by a dance specialist. It may include a creative focus, that might involve improvisation, creative exploration, creation of dance material and complete dances, enabling participants to contribute to choreographic and artistic decision making, sometimes leading to a live or film-based performance (often informal in nature) but not necessarily. Interestingly, in many cases in the literature, it seems “dance” is not defined.

Aesthetic contributions are also understood in their broadest sense. Returning to definitions from the likes of Osborne (1970, as cited in Smith-Autard, 2002) we suggest that aesthetics are made up of sensory, expressive and formal qualities which can be experienced, perceived and felt within dance. It is noteworthy that Smith-Autard argues there are rarely adequate words for these, which is perhaps why their contributions are so hard to grasp within Dance for Health settings. We also go beyond the idea of a “hierarchy of analytic aesthetic principles” to include more recent understandings of aesthetics as encompassing “embodied feeling” (Houston, 2015, p. 32), enmeshed with notions of fairness, justice, social action, suffering and community values.

Our understanding of artistic contributions is grounded in the idea that it is about coming to understand more about the art form itself. This will be different in, for example, contemporary dance, Bharatanatyam or Hip Hop. So, as Best (1992) states, artistic understanding is built on understanding how aesthetic principles coalesce or code meaning within different art forms or dance styles, making those art forms or dance styles what they are. But the aesthetic and artistic are not the same. Fancourt (2017, p. 68) defines the arts *in health* as (citing M. White & Hillary, 2009, p. 262) “creative activities that aim to improve individual or community health using arts-based approaches, and that seek to enhance healthcare delivery through provision of artworks or performances”. This is a more instrumental view than our understanding, describing what the arts offer as additional to the contributions of health professionals. Aside from Fancourt’s definition, the arts or “artistic” are rarely defined in the health literature, reflecting the lack of definitions of dance noted above.

We have maintained an open mind as to the varied possibilities for defining creativity given the huge range of definitions in the literature . We acknowledge the difference between big C (art-form changing) and little c creativity (everyday creativity) (Fancourt et al., 2019), as well as Vella-Burrows et al. (2017) articulation of “creative” “dance” as connecting to the human desire to add something culturally new, and to divergent and combinatory thinking. Wakeling (2013) offers thorough insight covering psychology definitions focusing on creative process, product and personality traits (e.g., Simonton, 2000); and we also draw on John‐Steiner (2000) and Amabile (2001) work on creativity as collaborative, communal and social; on democratic creativity (Banaji et al., 2010); on humanizing creativity where identity development is ethically entwined with process (Chappell, 2011); and on Camic et al. (2018) who provide a more material-socio-cultural definition of creativity. Creativity is perhaps therefore the best-defined concept within the literature and for the review we drew out a combined focus on the idea of newness, imaginative (divergent and combinatorial) thinking, individual-communal collaboration and connections to identity, value and ethics.

Our definition of “health and well-being” is delimited through a Western lens and culture of health which, alongside Western understandings of relationality and quality of life, includes a biomedical approach alongside psychological elements (Fancourt, 2017). As Sheppard and Broughton (2019) state, more recently what it means to be healthy in a Western sense also involves exploring varied approaches for quality of life across the lifecourse, hence the increasing inclusion of the arts. They also helpfully define wellbeing as a crucial aspect of health, drawing on Marmot (2011, p. 42) who describes wellbeing as “a multidimensional construct, which includes satisfaction with life, a sense of autonomy, control and self-realisation, and the absence of depression and loneliness”. We remained cognizant of various models of well-being including Deiner and Ryan (2009) Tripartite Model of Subjective Wellbeing; Seligman (2011) PERMA model of wellbeing; Ryff’s 6 dimensions of psychological wellbeing, as well as Deci and Ryan’s Self Determination Theory (Carter & Andersen, 2019). The review was open to dance health and wellbeing publications focused across the lifecourse, acknowledging that dance and health can be brought together at any time on life’s journey.

Finally, “dance for health” is a relatively newly adopted term to describe dance activity that takes place in various settings and has potential to positively impact health and wellbeing with the overriding focus on dancing not therapy. The International Association for Dance Medicine and Science (IADMS) describes how ‘dance for health “provides holistic, evidence-based activities for the individual to manage and adapt to physical, mental and social health challenges. In Dance for Health sessions, trained teaching artists engage people as *dancers*, rather than patients, in joyful, interactive, artistic activity.” (IADMS, 2020). We acknowledge this IADMS definition alongside emphasizing dance as an activity to engage in in its own right, within varied settings, with the capacity to contribute to health and wellbeing.

### Research aims and questions

Shaped by the above definitions, the aim of this paper is to review contemporary literature addressing the aesthetic, artistic and creative contributions that dance makes for health and wellbeing; as well as reviewing existing understanding of appropriate methodologies for investigating these. Specifically, it addresses the following key questions:

1. What are the aesthetic, artistic and creative contributions that dance makes to health and wellbeing across the lifecourse?
2. What methodologies (mixed/innovative?) are appropriate for investigating these contributions?

## Methodology

### Literature search strategy

Following the guidance on conducting systematic reviews (Petticrew & Roberts, 2005) and in line with studies in the field (Sheppard & Broughton, 2020), a literature search was conducted in Autumn 2019 for studies examining the aesthetic, artistic and creative contributions that dance makes to health and wellbeing across the lifecourse, and the methodologies appropriate for investigating these. This search included peer reviewed studies and grey literature (i.e., evaluation reports, unpublished studies and unpublished literature reviews). Grey literature was included in line with Paez (2017) stance on its value in systematic reviews allowing for all available evidence on a topic to be included. From our team’s expertise across practice and research, we are aware that the dance for health field is in its infancy and that grey literature often houses emergent understandings of aesthetic, artistic and creative contributions and more varied methodologies, also highlighting gaps in research and future research directions. These documents only occasionally find their way into peer-reviewed articles as their understandings and approaches are not always seen as “rigorous” in health journals. Yet, when these articles are viewed through, for example, a phenomenologically qualitative (Finlay, 2006), practitioner-research centred (Dowler, 2013) or posthumanist (Quinn & Blandon, 2017) lens, they offer credible and legitimate understanding of the contributions under investigation, which have the capacity to challenge current orthodoxy and publication bias (Paez, 2017).

Inclusion criteria were publications examining the aesthetic, artistic and creative contributions that dance makes to health and wellbeing across the lifecourse and/or methodologies for investigating these contributions. The search did not discriminate between dance styles. Exclusion criteria were 1) studies where the focus of the dance was not aesthetic, artistic and creative, 2) Dance Movement Therapy studies, 3) practices that define themselves outside of dance such as somatics and authentic movement. Results referring only to professional dancers and vocational training were also excluded.

**Figure 1.**
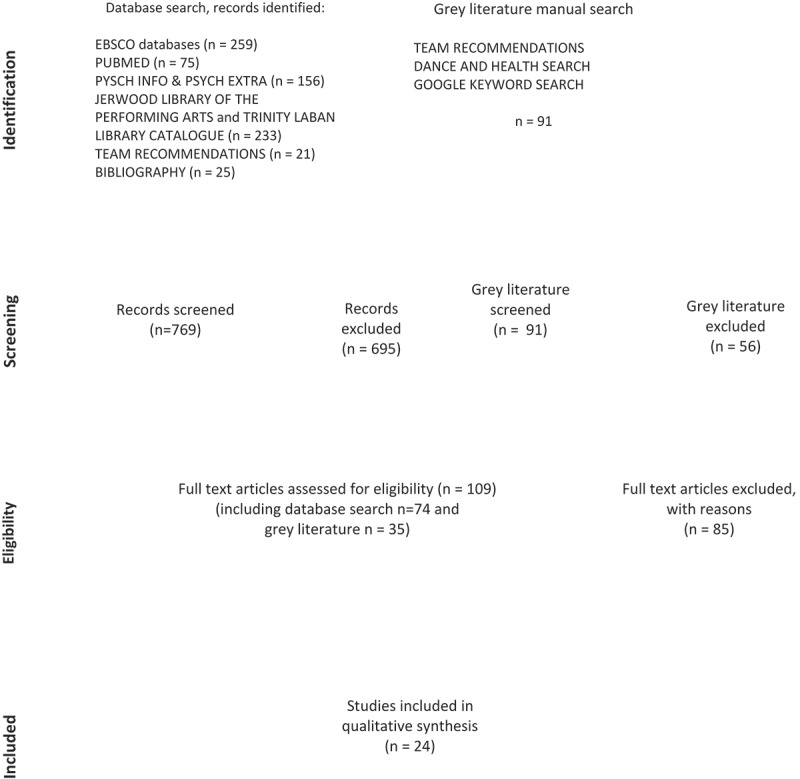
Flowchart of the literature identification and selection process

Figure 1 is a PRISMA flow diagram (PRISMA, 2015) showing the literature identification and selection process. The diagram has been modified to show the identification and screening of peer reviewed studies and grey literature separately before merging the results at the eligibility stage of the review process.

For peer-reviewed studies, a wide search was conducted on four databases, reflecting the transdisciplinarity of practice. Databases on health, education, psychological health, sports and performing arts were searched: EBSCO combined search on the British Education Index, Education Research Complete, Education Resources Information Centre, Medline and Sportsdiscus; Ovid search of PsychInfo and PsychExtra; and searches of Pubmed and the Trinity Laban Library Catalogue including the Jerwood Library of the Performing Arts. We combined the terms “dance”, “health OR wellbeing” and “creative OR artistic OR aesthetic”, including alternative term versions, plus the additional descriptors “creative movement”, “dance movement” “hip hop”, “ballroom”, “Laban”, “tango”. Other alternatives were removed since they did not return results. We restricted results to publications between 2000–2019 as this is the key period when dance for health work has developed; and to English language publications as this is the team’s working language and we did not have resource for translation to go beyond this. Limitations of the latter criteria are considered in the discussion.

The initial search yielded 723 results, to which we added references from research team members, and a manual reference search of relevant articles’ bibliographies, yielding an additional 46 references. Duplicates were removed and we conducted an initial screening of title and abstract for research question relevance, reducing the total to 109. These results were next categorized into sections, and records retained were those focussing on dance or dance plus one other artform, and cross-artform studies with significant creative, aesthetic, artistic or methodological interest, yielding 74 papers. References to articles focused exclusively on dance movement therapy (DMT) were excluded at this stage, since this therapeutic work is of a different type and derivation, with a medical purpose different to non-therapy defined dance for health, which is our focus here.

Our “grey search plan” used four approaches across 2000–2019 publications relevant to our field whilst aiming for “search sensitivity” (Paez, 2017, p. 234): resources and references suggested by the team, an internet search based on arts and health conference presentations, an internet search for organizations delivering dance or community projects with an identified health and wellbeing focus, and an internet search using keywords such as “dance health evaluation”. Initially the search looked at cross-artform studies before narrowing the focus to dance-only studies, yielding 91 results. Executive summaries, or full content where summaries were absent, were searched for key words and concepts relevant to our research questions. Studies with significant creative, aesthetic, artistic or methodological interest were retained, reducing the included grey literature to 35 studies.

### Rating and review procedure

These 109 papers were split between two researchers and each was rated twice, first according to their relevance to both of our research questions and secondly for methodological rigour, which was judged according to the publication’s research paradigm (for example, validity and reliability criteria for quantitative, and credibility and trustworthiness for qualitative publications) and the conventions of the publication format.

A 5-point rating scale was used of high, medium-high, medium, medium-low, and low. At intervals, papers were selected for cross-rating to ensure parity. Papers that rated high or medium/high for both our research questions and methodological rigour were included. These 24 papers comprised 11 peer reviewed articles, 3 PhD studies, and 10 evaluation reports (see Figure 2).
**Figure 2.**
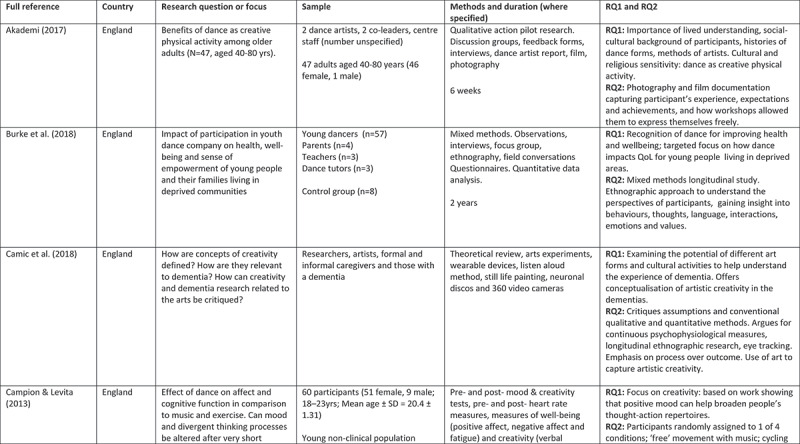
Detailed summary of results

**Figure 2.**
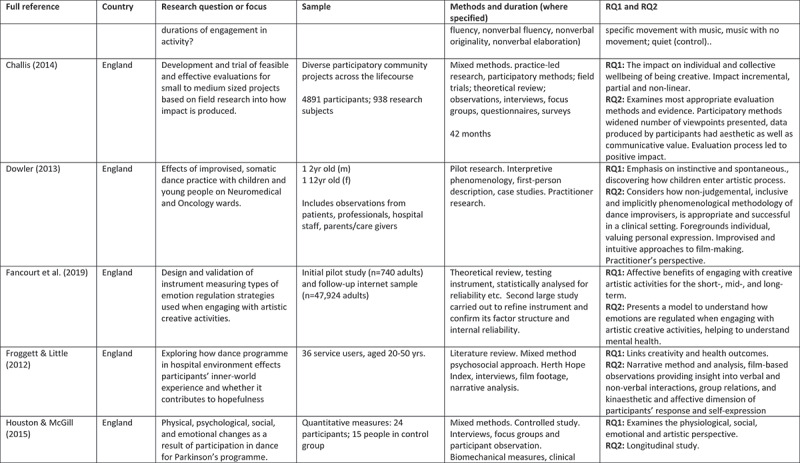

**Figure 2.**
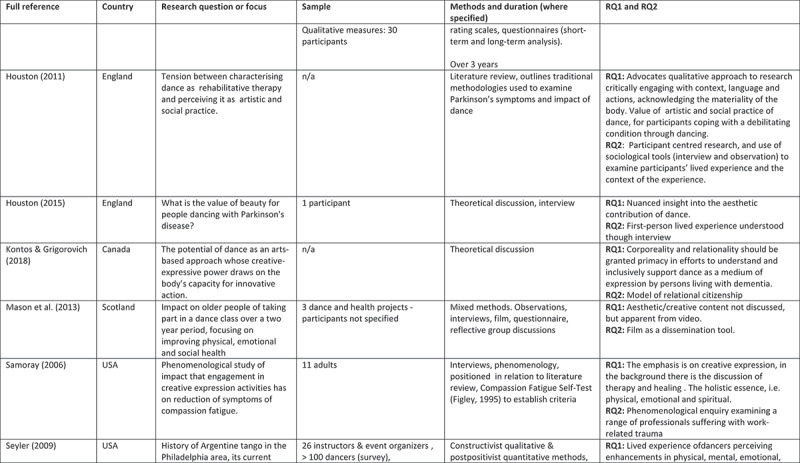

**Figure 2.**
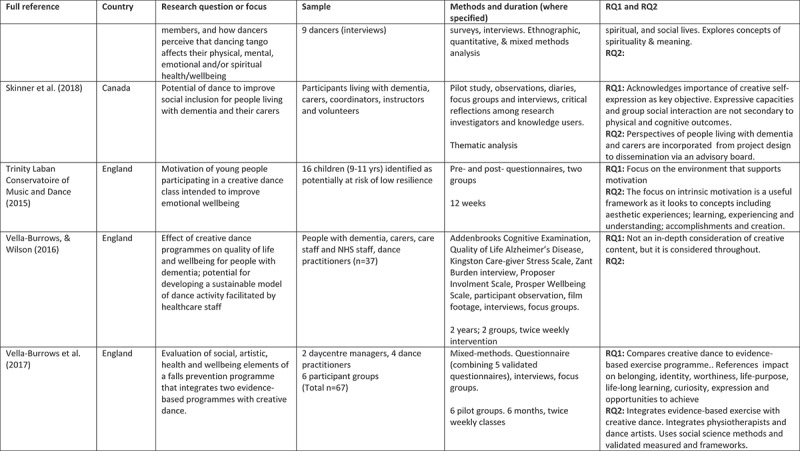

**Figure 2.**
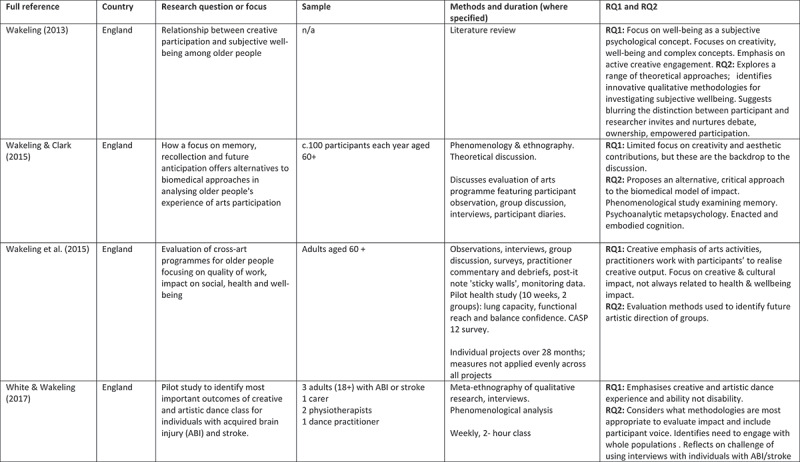

**Figure 2.**
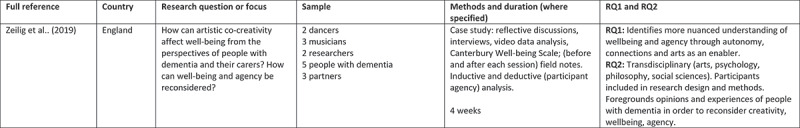

### Thematic analysis procedure

To conduct the qualitative thematic analysis (Braun & Clarke, 2006), the two researchers followed the standard process. They analysed article content framed by the review’s two research questions. Analytic stages included familiarization, coding, developing categories, then themes and reframing themes. Data was extracted for coding by close reading of all included papers. Midway through this process, codes were discussed and refined, developing groupings of categories and themes. This involved merging similar codes, and justifying whether unique codes should be included, renamed, subsumed or discarded. Towards the end of the analysis, categories and higher level themes were shared with two other researchers to further refine them. This process resulted in 7 main themes, each comprised of a different number of categories as discussed below.

## Findings

### Characteristics of included studies

Twenty of the included papers (83.3%) originated in the UK/England while two (8.3%) were from Canada and two (8.3%) from the US.

Five papers focused on dementia (20.83%) and three on Parkinson’s (12.5%). Eight papers addressed wellbeing for adults and for older adults (33.33%), including physical, social and emotional wellbeing. Three papers concerned health and wellbeing for young people (12.5%), including work in acute paediatric health (n = 1) and work on empowerment and resilience for disadvantaged young people (n = 2). The remainder of the papers focused on mental health (n = 1), falls prevention (n = 1), compassion fatigue (n = 1), acquired brain injury or stroke (n = 1), and how emotions are regulated through artistic creative activities (n = 1).

Ten papers (41.66%) did not specify the age range of participants or focused on health conditions dominant in older age. This makes commentary regarding the impact of dance across the life-course challenging. The remainder of studies spanned across the lifecourse with slightly greater weighting for older adults. This is in line with findings by Sheppard and Broughton (2020) who report a similar weighting amongst studies in arts and health.

Dance was the sole artistic focus in 15 of the 24 papers (62.5%), dance and music featured in five papers (20.83%) and cross-artforms were discussed in four papers (16.66%). Of the papers focused on dance or music and dance, 10 (41.66%) did not provide description of the dance practice, while the remaining papers focused on creative dance (n = 4), ballet (n = 2), contemporary dance (n = 1), somatics (n = 1), South Asian dance (n = 1), and tango (n = 1).

A range of research designs were employed across the included papers including mixed methods (n = 9, 37.5%), qualitative (n = 8, 29.16%), quantitative (n = 3, 12.5%), literature review (n = 2, 12.5%), and theoretical (n = 2, 8.33%). Sixteen of the papers used interviews as a research method (66.66%), nine used focus groups or group discussions (37.5%), and nine used standardized tests and scales (37.5%). Six studies utilized observations (25%), five used film (20.83%), five featured surveys or questionnaires (20.83%), three utilized diaries (12.5%), and two used evaluation forms or feedback surveys (8.33%). Other research methods included photography (n = 1), field trials (n = 1), creative methods (n = 1), post-it note “sticky walls” (n = 1), unstructured conversation (n = 1) and practice (n = 1).

Research Question 1: What are the aesthetic, artistic and creative contributions that dance makes to health and wellbeing across the lifecourse?

**Figure 3.**
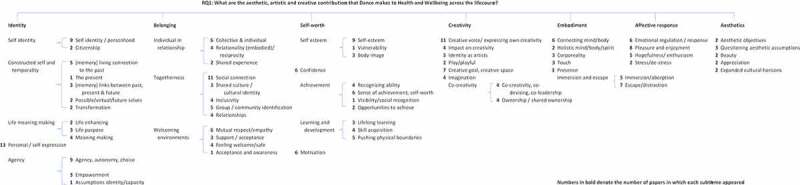
Main review themes for question 1

The main themes presented below (Figure 3) are ordered to reflect the number of papers that featured each sub-theme, which have been aggregated to create an analysis weighting.

### Identity

Our review articulates an active relationship between the development of self-identity, wellbeing, creativity, and dance. Under the overall theme of identity we have grouped concepts which include a more inward looking sense of self-identity, self-expression and meaning making together with a more public sense of ourselves as in artistic identity, cultural identity and issues of citizenship and agency. We found this theme in 21 papers (10 peer-reviewed, 3 PhD theses, 8 grey literature). According to Fancourt et al. (2019) new scale, “self-development” (including self-identity, self-esteem and agency) is a key strategy used to regulate our emotions when engaging in artistic creative activities.

Regarding *self-identity*, Dowler (2013) expresses a practitioner’s view of the need to bring “something of ourselves” (p. 165) to shared improvised dance, also reflected in research that finds dance has allowed participants to “reclaim humanity” (Houston, 2015, p. 31) or restore an “inner self” (Wakeling et al., 2015, p. 35). Two studies report that dance can bring different dimensions of the self together (Froggett & Little, 2012; Vella-Burrows & Wilson, 2016).

Other aspects of self-identity are invoked through dance, including taking on the identity of a dancer, linking with a sexual self and spirituality (Seyler, 2009) and linking everyday lives with cultural identity (Akademi, 2017). Zeilig et al. (2019) and Kontos and Grigorovich (2018) explore, embodied self-expression as part of a relational understanding of citizenship for people with dementia.

Several papers refer to *constructed self, temporality and meaning-making*, describing dance as a link to past or possible future selves. Five studies, all with older people, discuss a link to the past, including triggering memories, expressing reminiscence and re-awakening past selves through dancing. Other studies look towards the future, claiming dance helps establish healthy future habits amongst young people (Burke et al., 2018). Some address the future in a more transformative sense of imagining new possibilities (Challis, 2014), or changing attitudes through creative activity (Samoray, 2006; Vella-Burrows et al., 2017).

Seven papers found dancing was beneficial to participants’ wellbeing by enhancing life, by providing purpose in life, or as a meaning-making space. Reasoning behind these findings varied: for instance, Froggett and Little (2012) suggest dance is a place for meaning-making since it occupies an “in-between” space, between mind and body, bridging inner and outer experience, whilst Wakeling and Clark (2015) link it with the capacity of dance to link affective experiences in both past and present.

*Personal self-expression* through dance was discussed in 13 papers, connecting with wellbeing in different ways. Wakeling et al. (2015) linked personal expression to wellbeing within “self-realisation” which increased in their study amongst dancers with acquired brain injury, while Mason et al. (2013) linked it to the ability to express positive emotion. By contrast, Zeilig et al. (2019) found creative self-expression may be an uncomfortable experience, nevertheless linked to wellbeing through cultivating a feeling of being agential.

Dancing helped people feel more in control in their daily lives (Zeilig et al., 2019) enhancing their sense of agency in the world (Houston & McGill, 2015). Kontos and Grigorovich (2018) discuss the expressive power of bodies and their agential capacity, and “giving agency” was an explicit part of dance programme planning (Skinner et al., 2018; C. White & Wakeling, 2017). Several papers observed participants displaying agency whilst participating in dance, taking creative leadership (Dowler, 2013; Zeilig et al., 2019), or acting with autonomy (Wakeling et al., 2015).

#### Belonging

Belonging was identified in 17 of the 24 papers (6 peer reviewed, 9 grey literature, 2 PhD theses) in relation to the aesthetic, artistic and creative contributions that dance makes to health and wellbeing. The *individual in relationship to the collective* was often seen as meaningful; Froggett and Little (2012) identify a transitional and in-between status of individual and shared responses and of self, other and environment. Touch is identified as valuable for experiencing self and other, supporting meaningful social interaction and personal growth (Seyler, 2009). Support and solidarity amongst participants was seen to result in trust, care, inclusivity, positive interaction and intimacy (Seyler, 2009; Wakeling et al., 2015; C. White & Wakeling, 2017). Houston (2015) suggests shared expression of disability, alongside individual expressions, help appreciation of shared, unique ways of moving, important in linking aesthetics and health. Vella-Burrows and Wilson (2016) identify a self-reported rise in quality of life through relationships, not necessarily also reported by family carers; Burke et al. (2018) observed dance developed both positive peer relationships and some negative interactions.

The notion of *togetherness* comprises factors relating to social connection, shared culture or cultural identity, inclusivity, group or community identification, and relationships; Vella-Burrows et al. (2017) highlight recent interest “in the relationship between social identity and specific health behaviours” (p. 8). Several papers highlighted the importance of connection with peers by age or those with similar movement limitations (Froggett & Little, 2012; Wakeling et al., 2015). Other studies stress the supportive social environment (Akademi, 2017; Houston, 2015; Houston & McGill, 2015) and group contexts also helped participants develop personal and interpersonal skills (Burke et al., 2018). Skinner et al. (2018) and Seyler (2009) show the significance of non-verbal communication for connecting with others despite challenges.

In the papers, dance is associated with bonding and sharing different cultures as well as challenging attitudes as to what dance is perceived to be (Akademi, 2017; Froggett & Little, 2012; Seyler, 2009). Cultural inclusion extends to, and is supported by, the activity facilitators and other staff (Froggett & Little, 2012; Wakeling et al., 2015). Vella-Burrows and Wilson (2016) suggest that belonging/inclusion for those with dementia can be found through authentic and meaningful communication through creative expression and embodiment.

Identifying with, or belonging to, a group or community was characterized as a meaningful factor becoming increasingly important as programmes develop (Akademi, 2017; ;Houston & McGill, 2015 Mason et al., 2013). Houston and McGill (2015) demonstrate that the social isolation experienced by people with Parkinson’s makes feeling valued within a group particularly significant. Burke et al. (2018) argue that making connections in a reciprocally approving environment correlates to kinaesthetic empathy. Wakeling (2013) considers how social cohesion reinforces subjective wellbeing, which links to Seyler (2009) observations of building a tango community.

The notion of *welcoming environments* is a meaningful subtheme characterized by mutual encouragement and support (Burke et al., 2018) and facilitating independence, positive attitudes (Houston & McGill, 2015), familiarity and trust (Seyler, 2009). Vella-Burrows et al. (2017) identify how mutual respect and empathy create a welcome and safe environment where participants, including dance artists, do not feel judged. C. White and Wakeling (2017) demonstrate that mutual empathy created by dance results in caring for each other and trust; Zeilig et al. (2019) argue for an understanding of the role of vulnerability; and Seyler (2009) and C. White and Wakeling (2017) articulate how mutual respect inspires and invigorates.

#### Self-worth

Particularly in relation to the artistic and creative contributions that dance makes to health and wellbeing, the theme of self-worth was identified in six peer-reviewed articles, eight grey literature, and two doctoral theses. Concepts relating to this theme of self-worth and value are grouped under *self-esteem*, a sense of *achievement, learning and development*, and *motivation*. Fancourt et al. (2019) and Akademi (2017) highlight engagement with artistic creative activities improving self-identity, self-esteem and agency, as well as self-confidence linked to increased motivation. Houston and McGill (2015) and Mason et al. (2012) draw attention to lowered inhibitions and forgetting self-consciousness, although it is noted that lowered inhibition occurs over time (Vella-Burrows et al., 2017; Wakeling et al., 2015) and in later stages of dementia (Vella-Burrows & Wilson, 2016). Several papers correlate improved or more expansive movement with confidence or self-assurance (Burke et al., 2018; Dowler, 2013; Seyler, 2009), and opportunities for creative expression with confidence (Dowler, 2013; Mason et al., 2013; Seyler, 2009; Wakeling et al., 2015). Camic et al. (2018, p. 4) identify co-creativity as a process and tool for self-actualization.

A close link between self-esteem and self/body image is identified as improving outlook (Houston, 2015; Samoray, 2006). According to Zeilig et al. (2019), being vulnerable and empathetic enhances wellbeing and freedom for co-creation. For Wakeling et al. (2015) and C. White and Wakeling (2017), recognition of ability and higher self-expectations materialize through participant-led, or non-hierarchical, dance activities that are non-clinical, emphasize creativity, and are improvisatory, while Samoray (2005) links creative expression and self-awareness.

Notions of worthiness and *achievement* including feeling good, more capable, and loveable are reported to improve certainty about the future (Houston & McGill, 2015), while beauty and grace are seen as fundamental to identity, self-efficacy, and dignity (Houston, 2015). Burke et al. (2018) found positive feelings and emotions fostered through dance contributed to young people’s quality of life. The performative qualities of dance led to a sense of achievement (Froggett & Little, 2012), including positive perceptions and self-worth generated by the social recognition of public performance (Wakeling et al., 2015).

Dance is identified as an opportunity to achieve and take more risks (Akademi, 2017), through the intellectual challenge of *learning and development* (Seyler, 2009) as well as to gain or refine skills, particularly appreciated by older participants (Burke et al., 2018; Mason et al., 2013; Vella-Burrows & Wilson, 2016; Wakeling et al., 2015). For Burke et al. (2018), young people’s skill acquisition led to physical self-efficacy and recognizing capability, and, for Wakeling et al. (2015), it resulted in improved interpersonal skills and “reading” other people’s bodies.

Pushing physical boundaries is identified as important (C. White & Wakeling, 2017), for people with Parkinson’s (Houston, 2015; Houston & McGill, 2015) and for young dancers’ movement competency (Burke et al., 2018). Wakeling et al. (2015) show it connects to a strong sense of identity for older adults.

Houston and McGill (2015) identify a link between motivation and a supportive group for socializing. However, Camic et al. (2018) highlight assumptions are made about the motivation and engagement of participants with dementia; and Vella-Burrows and Wilson (2016) note a paucity of evidence concerning appropriate methods for engaging people with diminishing or inconsistent motivation. Trinity Laban (2015) focuses on how the dance learning environment, with facilitator feedback and delivery, effects intrinsic motivation, as well as communication development, teamwork and problem-solving skills.

#### Creativity

Given our research questions directly concern creativity, it is not surprising that this theme appeared in all reviewed papers (11 peer reviewed articles, 3 PhD studies, and 10 evaluation reports) either as an aim/process, method or outcome. In several studies of existing practice, “creativity,” and enabling participants to develop or express their own “creative voice,” were key aims. Sometimes this was presented by contrast to focusing primarily (or at all) on “therapy” or “disease” (Houston, 2015; Kontos & Grigorovich, 2018; Skinner et al., 2018; C. White & Wakeling, 2017).

“Creativity” as a theme is necessarily interwoven through the review, linked variously to different health and wellbeing outcomes. For example, developing agency, as described above, was linked to developing participants’ own creative voices, and to the emergence of creative self-identity, a discussion Houston (2015) skilfully situates within the wider field of disability and community dance. The *development* of creativity was an explicit aim or outcome in some project evaluations (Akademi, 2017; Wakeling et al., 2015) and research studies: Campion and Levita (2013) studied the impact of dance on creativity, finding an increase in creativity amongst a young non-clinical population. Vella-Burrows and Wilson (2016) map levels of creative expression in dance participants with dementia.

Different creativity dimensions feature in different studies. Camic et al. (2018) and Zeilig et al. (2019) both describe playfulness as characteristic of creative work they observe with people with dementia. Others focus on imaginative engagement: for example, Froggett and Little (2012) found that in an acute mental health setting, imagination was engaged in the process by which inner experiences were translated into movement.

Co-creativity was explored by Camic et al. (2018) and Zeilig et al. (2019), both articles stemming from the same research programme and thus adopting a similar concept. They describe creativity as dialogic, foregrounding relationality, process and experience, and moving away from a reported cognitive bias in creativity research. Other studies observe similar characteristics of practice, describing them as collaborative/interactive creation (Seyler, 2009), and co-ownership of the artistic product and process (Akademi, 2017; Wakeling et al., 2015).

#### Embodiment

Embodiment was identified as a meaningful characteristic of dance’s aesthetic, artistic and creative contributions to health and wellbeing in eight peer-reviewed, five grey-literature papers, and three PhD studies, perhaps most in relation to aesthetic sensing/feeling. Different dimensions included increasing body awareness (Wakeling et al., 2015), and *connecting mind and body* through dance (Dowler, 2013; Froggett & Little, 2012). Evaluation reports also found the *holistic, mind/body/spirit* dance benefits were valued (Akademi, 2017; Burke et al., 2018). Dance is described as an embodied expression of emotional experience, especially in dementia settings (Kontos & Grigorovich, 2018; Vella-Burrows & Wilson, 2016).

Wakeling and Clark (2015) explore meaning-making’s *corporeal* character. Sensory descriptions in accounts of dancing echo this corporeal understanding (Seyler, 2009). Kontos and Grigorovich (2018) also identify intercorporeal creative engagement in dementia care.

The notion of “presence” related to *touch*, has an embodied quality (Dowler, 2013; Houston, 2011; Vella-Burrows & Wilson, 2016). For example, practitioner-researcher Dowler (2013) describes creating a steady presence through touch, using it as a meeting place between individuals. Skinner et al. (2018) also note touch as an opportunity for connection, although one evaluation describes deliberately avoiding physical contact, allowing participants to connect whilst still giving each other space (Akademi, 2017).

Two complementary embodiment subthemes were *immersion and escape*. Both are reflected in Fancourt et al. (2019) analysis of emotional regulation strategies for artistic creative activities. “Immersion” in our analysis refers to dance as something within which participants could become absorbed (Froggett & Little, 2012; Seyler, 2009), similarly drawing on an embodied creative experience of Csikszentmihalyi’s “flow” (Challis, 2014; Vella-Burrows & Wilson, 2016). Several papers found this to be a means of “escape” from difficult emotions or everyday realities. For example, Houston (2015), theorizes that beauty has the potential for altering perspective through embodied feeling in dance, taking us away from our usual priorities.

#### Affective response

Affective response, related to all three foci (aesthetic, artistic and creative) was identified as meaningful in 15 of the papers (6 peer review, 7 grey literature, 2 PhD theses). That artistic creative participation leads to *emotional regulation* is identified as including stimulation and happiness (Akademi, 2017), positivity (Burke et al., 2018), significant improvements in emotional wellbeing and fatigue reduction (Campion & Levita, 2013), improved cognitive function and wellbeing (Houston & McGill, 2015) and emotional growth (Seyler, 2009).

Several papers identify a positive correlation between dance and emotions, identifying *pleasure and enjoyment* as meaningful (Akademi, 2017; Froggett & Little, 2012; Houston, 2015; Houston & McGill, 2015; Seyler, 2009; Vella-Burrows & Wilson, 2016; Wakeling et al., 2015). Dance’s energizing effect is characterized as a valued distraction (Houston & McGill, 2015) and dancing results in feeling more alive and liberated (Mason et al., 2013).

Several studies found dance increased *hopefulness and enthusiasm*, including facilitating a positive future view (Froggett & Little, 2012), leading to hope, improved future certainty and the realization that life is still worth living (Houston & McGill, 2015; Vella-Burrows & Wilson, 2016). How creative activities ameliorate *stress* is noted by Samoray (2005) and Burke et al. (2018). Samoray (2005) identifies creativity as an escape mechanism that can boundary and contextualize grief. Burke et al. (2018) indicate that dance can help young people to cope with day-to-day difficulties, enhancing their wellbeing. However, they suggest dedication to an activity can result in “tensions and conflicts with an already busy life” (Burke et al., 2018, p. 15).

#### Aesthetics

Again, given our concern with aesthetics, it is not surprising this theme appeared, although as opposed to creativity, only in seven papers (3 peer-reviewed articles, 2 doctoral theses, 2 grey literature). Peer-reviewed papers foreground the *aesthetic objectives*, processes, or traits of dance (Camic et al., 2018; Houston, 2015; Wakeling & Clark, 2015). Challis (2014) and Houston (2015) identify how the inclusive and democratic nature of community dance *questions aesthetic assumptions*, valuing the individual dancer and resisting commodification. Houston (2015) highlights a link between aesthetics and health, discussing how dance’s aesthetic focus reclaims ideals of *beauty* and grace, which counter typical discourse on Parkinson’s. Wakeling et al. (2015) correlate body aesthetic with confidence.

Participation is linked to increased *appreciation* and *expanded cultural horizons* by way of deepening understanding of dance (Seyler, 2009), appraising one another’s work, and developing a common artistic and aesthetic language (Wakeling et al., 2015). Belonging to an arts organization and high-quality provision tailored to participants, but without emphasizing living with Parkinson’s, is identified as important by Houston and McGill (2015). Wakeling et al. (2015) find participation leads to widened appreciation for dance and other art forms, while group diversity widens cultural experience.

Research Question 2: What methodologies (mixed/innovative?) are appropriate for investigating these contributions?

**Figure 4.**
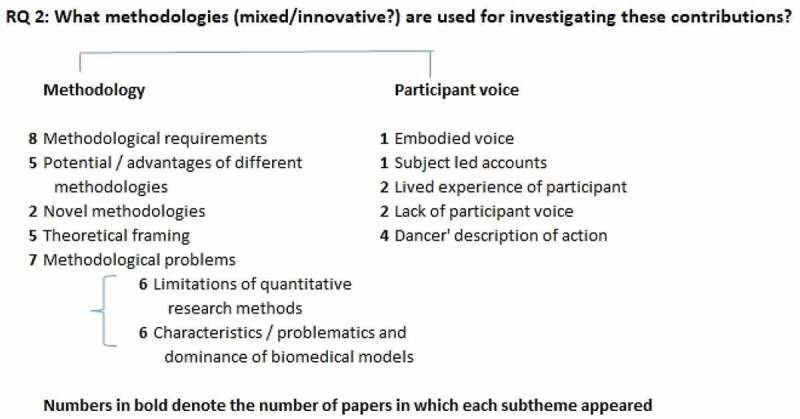
Main themes related to question 2

On the whole, there was less material to draw on in answering the second of our research questions. Five of the papers focussed extensively or exclusively on methodology, so the data presented is weighted towards these (four peer reviewed, one PhD study focussed on evaluation methods), supplemented by short sections from other papers. This data was analysed into two themes: methodology, and participant voice (see Figure 4).

## Methodology

Of the papers included for their investigation of dance’s aesthetic, artistic and creative contributions to health and wellbeing, a majority (n = 16) use either a qualitative or mixed methods approach, with only three studies exclusively collecting quantitative data. A strong critique of quantitative methods is provided in several of the papers, and of a reliance on a biomedical model of health often allied with this methodology. Other methodological problems and weaknesses in the field are also described, and consideration is given to the particular requirements for researching creativity, dance, and health.

The *methodological requirement* to capture the complexity of the relationship between dance and wellbeing/health was identified in four papers, all viewing dance as a complex social, artistic and creative activity, involving rich and diverse processes of meaning-making (Camic et al., 2018; Froggett & Little, 2012; Houston, 2011; Wakeling & Clark, 2015). Fancourt et al. (2019) also identify the need for understanding complexity, addressing this through developing a means to quantitatively capture the nuanced inter-relating detail of emotional regulation strategies. Houston (2011) identifies a need to reflect the embodied qualities of dance within the methodological approach. Challis (2014) and Camic et al. (2018) both highlight the need to emphasize process rather than outcomes in creativity research. It might be argued that these notions of capturing complexity, reflecting dance’s embodied qualities and emphasizing process over outcome are at the heart of necessary future methodological developments in this area.

Six of the studies (five peer-reviewed, one evaluation) highlight the *limits of quantitative methodologies*. In the context of studying aesthetic, creative and artistic dance elements, these are criticized for not reflecting the richness of the dancing experience (Houston, 2011; Vella-Burrows & Wilson, 2016), nor recognizing self-expression and social interaction through dance (Skinner et al., 2018). Froggett and Little (2012) and Camic et al. (2018) describe the difficulties of measuring specific changes within the complexity of dance programmes. Zeilig et al. (2019) highlight a lack of consensus in the field on how best to measure wellbeing.

There is also criticism from Zeilig et al. (2019) that a *biomedical paradigm* can be dehumanizing for research participants, positioning them in a passive role; this links to claims that a biomedical approach compartmentalizes the body and focuses on symptoms and treatment, rather than on the whole person (Camic et al., 2018; Dowler, 2013; Houston & McGill, 2015; Kontos & Grigorovich, 2018). Positioning dance as a therapy or treatment can also make it hard to recognize its artistic qualities (Houston, 2011, 2015).

The same authors claim qualitative research has better potential for studying the creative, embodied and artistic dimensions of dance, with more emphasis on the individual’s dancing experience, together with an emphasis on complexity rather than reductionism (Froggett & Little, 2012; Houston, 2011).

A number of *other methodological problems and weaknesses* are identified. Particular theoretical framings are problematised—for instance, both Camic et al. (2018) and Kontos and Grigorovich (2018) criticize a cognitive bias in researching creativity with people with dementia. Challis (2014) finds a lack of consensus on what constitutes “good evidence” of the impact of creativity on wellbeing in evaluations, also finding the data produced using creative methods was not necessarily acceptable to the audiences for evaluation. Zeilig et al. (2019) note that, for those living with dementia, there is little consideration of participants’ views on the arts research methods they are involved with, and this may also be true of other research populations.

While individual papers make claims for the *benefits of different methods*, no specific thematic pattern emerged. Froggett and Little (2012) advocate a mixed method approach including interview data, film and participant observation in order to capture emotional, physical and affective responses; Dowler (2013) argues for a practitioner-researcher model building both theory and embodied knowledge; Houston (2011) advocates using Laban Movement Analysis to combine the in-depth embodied knowledge of dance studies with a focus on individuals’ experiences. Creative methods are praised for producing new data that may challenge the status quo (Challis, 2014) and for supporting public engagement in research (Camic et al., 2018). Camic et al. (2018) also describe a number of novel methodologies, such as wearable data collection devices and 360-degree video cameras which collect detailed psychophysiological data and group interactions.

### Participant voice

The importance of “participant voice”, as both method and as part of co-creating the research, was identified in eight peer-reviewed articles. Houston (2011) points to researchers’ responsibility to individual participants’ embodied voice and response. Dowler (2013) recognizes participants’ lived experience through interpretative phenomenology, while Skinner et al. (2018) achieve this by adopting advisory boards involving what they refer to as key stakeholders to guide the research. Kontos and Grigorovich (2018) note that focusing on objective measures and proxy testimonials neglects the first-person experiential perspective of participants living with dementia, while Camic et al. (2018) argue that defintions of creativity fail to account for participants’ voice. Wakeling and Clark (2015) highlight the need to unpick the nuance and complexity of experience through subject-led accounts.

Several papers use descriptive and analytic frameworks from dance to illuminate intention, response, communication and expresssive capacity. Froggett and Little (2012) describe physical and attentional changes in participants to characterize how physical engagement impacts state of mind. Dowler (2013) contrasts the embodied knowledge of somatic practitioners and the perspectives of participants, patients and medical practitioners by highlighting the nuanced qualities of intentional touch. In addressing the methodological challenges of research into dance for people with Parkinson’s, Houston (2011) argues that the approach and expertise of dance researchers, which includes methods of movement analysis, is needed in order ‘to focus attention on the dancing person, rather than merely his or her disease (p. 330). Houston’s field notes articulate the joy of moving, and the humanizing effect of dancing with others (2015). Describing the dancing experience from the perspective of the participant or utilizing dance-researcher observational expertise eludicates dance as a complex embodied phenomenon.

## Discussion

This review was instigated within a collaborating team of 20 years standing, in response to our identifying a twin gap as to understanding: the artistic, aesthetic and creative contributions of dance to health and well-being across the lifecourse; and appropriate methods for researching this. The review’s ensuing outcomes offer key insights into the former, but less into the latter. We begin the discussion by considering the limitations of the articles included in the review.

### Limitations of research reviewed

In the first instance we need to be cognizant that the review excludes high quality work written in other languages than English, and even within the English language remit features only three countries. Other limitations include that, whilst terms like “aesthetic” and “creative” are often well-defined (potentially explaining why they have emerged as review themes), articles are lacking in definitions of “dance” and “artistic”. The terms creative, artistic and dance are easily conflated in circular definitions. Whilst we have already referred to the IADMS definition of dance for health, the relationship between these two concepts is also rarely defined in the review papers. The representation of research from different periods of the lifecourse is also patchy with age range sometimes not defined. Because of the small number of papers available, coverage of health conditions is also inconsistent.

Methodologically, there is a lack of consensus on how to measure concepts like wellbeing, possibly due to the number of different models and theories of wellbeing (e.g., Ryff‘s multidimensional model of psychological well-being; Keyes’s theory of Flourishing; Seligman’s PIRMA-theory) (Carter & Andersen, 2019). Lastly, there are challenges when trying to decipher some of the research studies published because of unclear descriptions of the dance interventions themselves. This has been noted by others (e.g., Fortin, 2011) and is perhaps due to the focus being firmly placed on outcomes over process, as well as perhaps demonstrating a difficulty in having the language to talk about dance per se.

## Overall contribution of reviewed research to answering research questions

### Discussion regarding question 1

Overall, the 24 papers offer a rich insight into the artistic, creative and aesthetic contributions that dance can make to health and wellbeing clustered around seven main themes: identity, belonging, self-worth, creativity, embodiment, affective response and aesthetics. Whilst we have represented the themes in a tree diagram, they do not exist alongside each other in isolation. A three-dimensional fluid sculpture might perhaps offer a better insight into their inter-relationship. The analysis showed fluidity and entanglement between theme boundaries with, for example, identity and belonging closely related, as well as embodiment, affective response and aesthetics being clearly intertwined. Lower level categories such as meaning-making, the ability to change the future, presence and touch might be seen as interconnecting threads between higher-level themes. These interconnections offer us more nuanced insight into the contributions that dance makes to health, some of which which may appear to be “in the moment”, but have the potential for lasting impact. Here, our findings are in agreement with Fancourt (2017) who points out that it is hard to understand the world of arts and health in terms of models of relationships between the two since the breadth of arts impacts make it difficult to “pin down” and evaluate in a traditional sense. Both Fancourt’s work and this review perhaps indicate a need to accept the complexity of dance’s contribution to health in order to understand and research it appropriately.

Several of the categories highlighted in the analysis can to some extent be explained by psychological theories and models. These include categories such as belonging and self-worth, self-esteem, confidence, social interaction and personal growth. Deci and Ryan’s Self Determination Theory argues that people grow and function optimally in society when three basic psychological needs are met. These needs are relatedness—the universal want to interact with and be connected to others; autonomy—the universal need to be causal agents of one’s own life; and competence—a perceived feeling that an outcome can be mastered (Deci & Ryan, 1985). Self-determination theory considers well-being as not simply reflecting the experience of positive affect. Quested and Duda (2009, p. 11) point out that “the emphasis is on understanding the motivational factors leading to personal realization and optimal functioning (i.e., eudaimonic rather than hedonic well-being).” Our findings indicate the potential of dance to cultivate the areas of wellbeing described by both Quested and Duda (2009) and Deci and Ryan (1985).

Other categories that emerged from our analysis, such as meaning-making and the ability to change the future, connect well with models of wellbeing. The PIRMA Model wellbeing theory (Seligman, 2011) for example, identifies five essential elements of wellbeing: positive emotions, engagement, positive relationships, meaning and achievement/accomplishment. These elements of wellbeing are mentioned either directly or indirectly in almost all the papers in our review further supporting the impact of dance.

Delving further into the findings, there are nuances worthy of discussion. The dominance of identity as a theme is palpable (present in 21/24 papers). This has been a growing focus in dance for health research (e.g., Lussier-Ley & Leger, 2011). Perhaps because of the dominantly psychological approach in this research to date, many of the papers articulate identity as “self” identity with self engaged in relations, reflecting approaches which centralize the individual. There is less of a sense of identity as entangled with the environment and others, for example, following more sociologically or posthumanist driven understandings. There are hints within the review of relationships between the constructed self, temporality and meaning making (e.g., Burke et al., 2018) indicating further potential for considering dance for health across time/the lifecourse rather than as an intervention prescribed to solve an immediate problem. It is worth noting that where previously changes in identity might have been reported as anecdotal, here 21 papers confidently include discussion of it within their evidence base.

Touch is included as a theme within belonging, although, as noted above, it features in smaller ways in other themes too. Touch (especially non-clinical/non-care related) and the in-between space (between individual and group) emerge as highly relevant factors in how dance contributes to health artistically, aesthetically and creatively. The review shows that these factors are seen as an overlooked means to understand self and other, feed social interaction, allow for vulnerability and build trust, all vital as part of healing and ameliatory responses to long-term health conditions. The kind of belonging created here also relates to how different aesthetics to those promoted in performatively-based dance can be forefronted and valued (e.g., Houston, 2015), and emphasizes the dance practitioner/artists’ own belonging in a more equal rather than hierarchical way, alongside participants.

Unsurprisingly, the categories self-esteem and self-confidence were often conflated in our analysis. While this is perhaps because of participants’ understanding of these terms, our analysis indicates that dance can positively impact both self confidence defined as one’s belief in one’s abilities and self-esteem defined as one’s sense of one’s worth. Motivation also appeared in several places within our analysis, again not always clearly defined. Nonetheless our analysis indicated that dance has the potential to foster participants’ intrinsic motivation.

Creativity came through as a relatively strong theme, reflecting its inclusion as a key term for the review. A standard type of creativity research is included, measuring the impact of dance on creativity (e.g., Campion & Levita, 2013). Creativity is also defined and researched not just in relation to creating the “new” but as connected to voice and expression, and research explores how these contribute to health (e.g., Vella-Burrows & Wilson, 2016). Authors also propose new ways of understanding creativity through their work with different populations, e.g., Camic et al. (2018) research with people living with dementia. Overall, the idea of co-creativity emerges as a strong new area (e.g., Zeilig et al., 2019), again moving on from more cognitive, individualized accounts prioritized previously, reflecting a move in this direction in creativity research more widely (e.g., Chappell, 2018; Glăveanu, 2013; John‐Steiner, 2000).

Within less dominant themes, there are some examples of fledgling developments. For example, the complexity of the connection between beauty, embodiment and wellbeing is dealt with effectively by Houston (2015). This exploration of the aesthetic makes up a relatively small part of the review papers’ emphases. Whilst we have identified it as a part of wider discussions about dance’s contribution, the actual amount of work in this area is relatively low. Wakeling’s cluster of papers within the review offers connected insight into how dance-based artistic contributions can change participants affectively through their emphasis on embodiment, as well as expanding cultural horizons, all developing wellbeing. Also in relation to the affective, there is insight into how elusive elements such as feeling more alive and liberated, particularly through engaging in non-habitual movement patterns, can be beneficial for rehabilitation (Dowler, 2013). Zeilig et al. (2019) offer an interesting re-framing of wellbeing as well as creativity, emphasizing embodiment and relationality, closely linked with agency, contrasting with previous, more individualized accounts. All of these fledgling theorizations present fresh new starting points for further investigation.

#### Discussion regarding question 2

Methodologically, the majority of the 24 papers argue for the use of qualitative or mixed methods as appropriate to understanding aesthetic, creative and artistic contributions of dance to health; only three use entirely quantitative means. This emphasizes that despite a qualitative dominance, there is no “right” research approach in this area—a view supported by others (eg. Clift et al., 2019; Fortin, 2011). Most important is the type of research question under investigation, the epistemological and ontological position and argument of the authors in relation to their area of study, and ensuring that appropriate rigour criteria for the paradigm are employed. The questions in the papers included were often aiming to characterize or understand the complexity of interactions.

Methodological questions are also raised across the papers about how to best capture elusive elements like embodiment; the review draws these together offering insight into current best practice in this area. Some authors tackle this through more traditional phenomenological approaches prioritizing subject led accounts (Samoray, 2006; Seyler, 2009; Wakeling & Clark, 2015). A small but growing number of others advocate new technological methods, including for example: photography, film (Akademi, 2017); neuronal discos, 360 cameras and wearable devices (Camic et al., 2018); practice-led approaches where data produced by participants had aesthetic as well as communicative value, e.g., meditative mark-making, expressive mark-making, video and mapping collage (Challis, 2014). Alongside this are calls to offer both dance artists (with all their embodied expertise to analyse and interpret movement-based developments) and participants roles beyond the passive and to engage them, and their embodied voice, more in research data collection and protocols (Zeilig et al., 2019), also connecting to efforts to move beyond a cognitive emphasis. These perspectives from review papers reflect a move in wider arts for health research towards using arts approaches (Fraser & Sayah, 2011), and new approaches (Choo et al., 2019) including philosophical analysis techniques in the vein of the rhizomatic approach of Deleuze and Guattari (e.g., Atkinson & Scott, 2015) and valuing practitioner reflective practices focused on elements like dialogic, kinaesthetic empathy (Wakeling, 2014).

## Conclusions and implications

In this last section of the article we pull together the contributions to knowledge that the review offers and provide a foundation to shape the research agenda going forward. Overall, whilst acknowledging the limitations of this fledgling field and the conceptual and methodological challenges it faces, this review offers seven carefully-synthesized, rich themes demonstrating valuable contributions that dance makes beyond the physiological. Prior to the review these themes remained scattered across publications; with them collated together here, we can pinpoint the best of our knowledge in this area at this time and provide a framework for our professional community to more confidently shape future research and evaluation. We propose that future research should explore further, how dance impacts health and well-being through the 7 themes identified in this review: identity, belonging, self-worth, creativity, embodiment, affective response and aesthetics, *and* the complexity of their interaction through connecting themes such as meaning-making, the ability to change the future, presence and touch. These latter elements especially may appear to be ephemeral and to date be considered difficult to research or measure, but this review synthesis demonstrates their “research-ability” with appropriate methodologies and centres them more clearly for future research attention.

Building on this, the review shows that further consideration needs to be given to mixed methodology studies, and how concepts like wellbeing might be more complexly measured whilst being complemented by qualitative data collection. This would especially build on Fancourt et al. (2019) research, applying their newly developed scales such as the Emotion Regulation Strategies for Artistic Creative Activities Scale in varied contexts.

However, the review also proposes new epistemologies and methodologies including from phenomenology, the critical theory of the likes of Deleuze and Guattari and posthumanism; and related methods such as the use of film and arts-based data collection; alongside including dance artists/practitioners and participants as co-designers and/or voices within research). We suggest that colleagues may start to use these more, which will lead to their value being articulated in relation to more traditional quantitative/qualitative and mixed methods. This will involve delving deeper into questions of underlying epistemology and ontology to interrogate assumptions as to the appropriateness of dominant paradigms such as quantitative and even qualitative approaches—which are perhaps not being heavily enough critiqued currently.

Related to this, Fortin (2011) discusses Coles’ conceptual framework (2005) as useful in understanding the field of dance and health since it acknowledges the tensions between a medical model of health and a social/ artistic one (Fortin, 2011). She cautions us not to overly rely on a particular way of knowing in order to ensure a balance of both qualitative and quantitative dance for health research. By offering the synthesized themes and related methodological suggestions, this review contributes to the conversation between stakeholders (including academics, health and arts professionals, policymakers and participants) as to which methodologies we use when, what their underpinning philosophical assumptions are, and the claims it is therefore appropriate to make from them. If this work is to continue to build, we argue that part of this conversation will need to focus on creating space for new methods, and understanding of appropriate rigour criteria. As with our *Dance, Health and Wellbeing: Debating and moving forward methodologies* project, these conversations would benefit from engaging with and hearing both policymakers, and also participants and practitioners.

Looking forward, alongside the future conceptual agenda and new methodologies offered above, there are a number of implications and recommendations from the review, which further contribute to the field and will aid its development. The review indicates that it is time to expand the geographical, lifecourse and health condition scope of this kind of research, which might in turn contribute to a less problem-solving, more ongoing lifecourse approach to the relationship between dance and health. As a community, we should aim to create space to consider and define the nuances of what we mean when using terms like “dance”, “artistic” and “dance for health”, as definitions of these key terms were consistently lacking in the literature reviewed. There is also further work to be done to delineate the distinctions and intersections between the concepts of embodiment, artistic and aesthetic. Whilst the latter two terms may often be aligned we need to better understand what inclusion of the “aesthetic” brings alongside but also additionally to the artistic, and how both of these relate to embodiment. Gaining clarity on this, whilst acknowledging what their complex interactions add to impacts, will influence how each of these terms is prioritized in future research. Also, attending to Fortin (2018, p 152) point that there is “a need to value and assess the process (the dance intervention) as much as the product (the results of the research)” could well help to develop these definitions.

The review suggests that it is timely to extend theorizing of concepts like identity and creativity beyond individualized understandings, seeking to encompass more dispersed theorizing. These were mentioned in 21/24 and 24/24 publications respectively, so are clearly priority areas. There are inklings of this in relation to creativity in the review, and in the work of colleagues such as Quinn and Blandon (2017) within music for health work, but there is certainly space within dance for much greater consideration of these perspectives. This potential for relational identity and co-creativity as key research areas gels with Fancourt & Finn, 2019, p. 18) comment that “While there is no consensus that any one type of arts programme is the most effective, results appear to be strongest when individuals and communities are actively involved in the creation of the art”. The review indicates that involvement in relationally-driven dance creation has the potential to subtly contribute to well-being. Ensuing connections to and impact on mental health is an area ripe for further research.

Where the review shows small pockets of understanding are developing in relation to areas such as aesthetic contribution, these would benefit from expansion, including building on the idea in a number of publications that dementia settings should not be viewed as in “deficit”, but as able to positively extend our understanding of what it means to be creative. Another area for focus here is the role and voice of dance artists and practitioners themselves and what we might learn from them about researching and evaluating dance’s more ephemeral contributions. This connects to a wider point that dance interventions are non-clinical, raising questions as to what artistic practice contributes to health. These questions begin to be answered in the seven themes articulated above, via categories such as touch, affect, presence, vulnerability and hope, to name a few, which are perhaps uniquely generated by the combined physicality, relationality, artistry and self expression that dance entails. This acknowledgement of dance’s unique combination of physicality, relationality, artistry and self expression can go some way to addressing the inadequacies of definitions of dance and artistry highlighted by the review. Understanding this combination is perhaps key to developing research which defines these terms, allowing for better articulation of related epistemologies and ontologies and accompanying appropriate methodologies and methods. As rigorous research, such as that in this review, begins to increasingly capture these elements, dance health researchers can more confidently complement traditional health measuring, to identify mechanisms or processes which may lead to longer term impacts “under the measurement wire”.

It is worth noting that within the timeline of our review, Clift et al., 2019) has written to critique the self-congraulatory nature of some dance for health publications which do not always focus on methodological rigour and can make assumptions which are not fully grounded in evidence. Whilst the professional community moves forward with recommendations from this review, it will be wise to heed his inherent warning. However, as a means to combat his concerns, our review alerts colleagues to the need for appropriate claims from appropriate methods. We argue that the review also shows the complexity and nuance of the best current, rigorous and varied methodological research into the aesthetic, artistic and creative contributions that dance makes to health, and articulates a clear research agenda for colleagues going forward .
